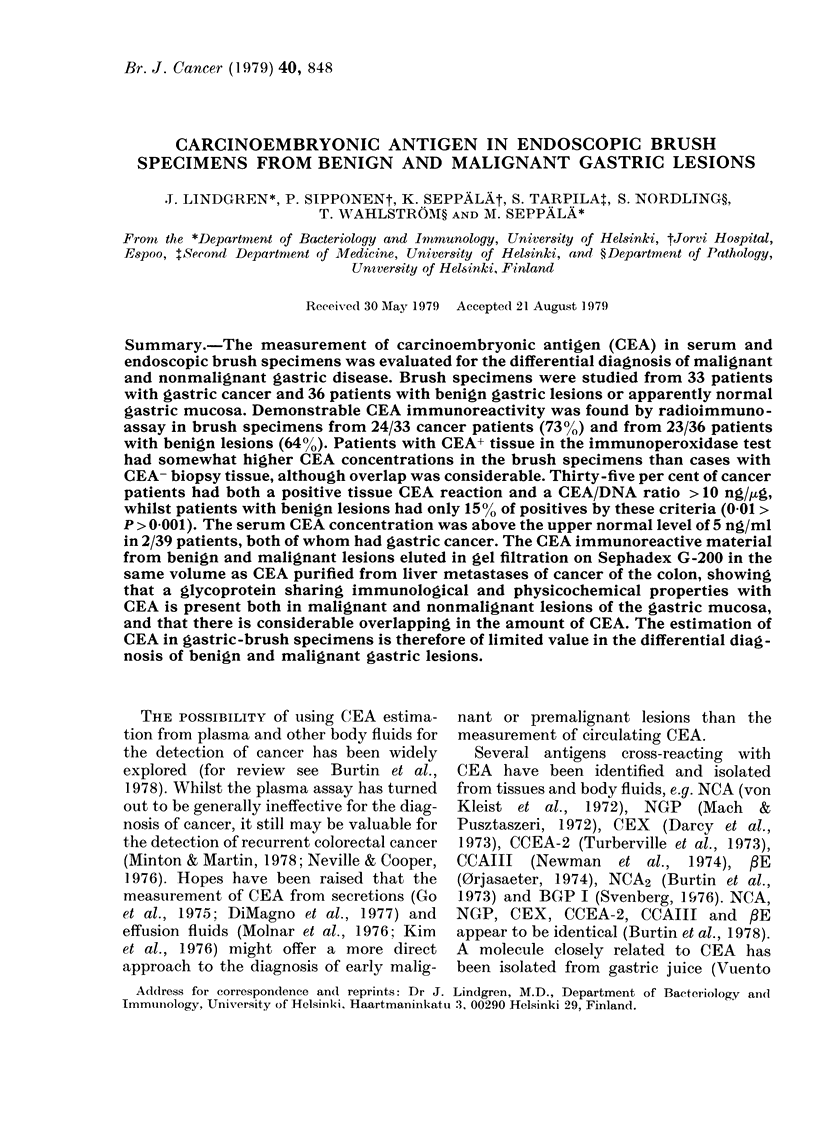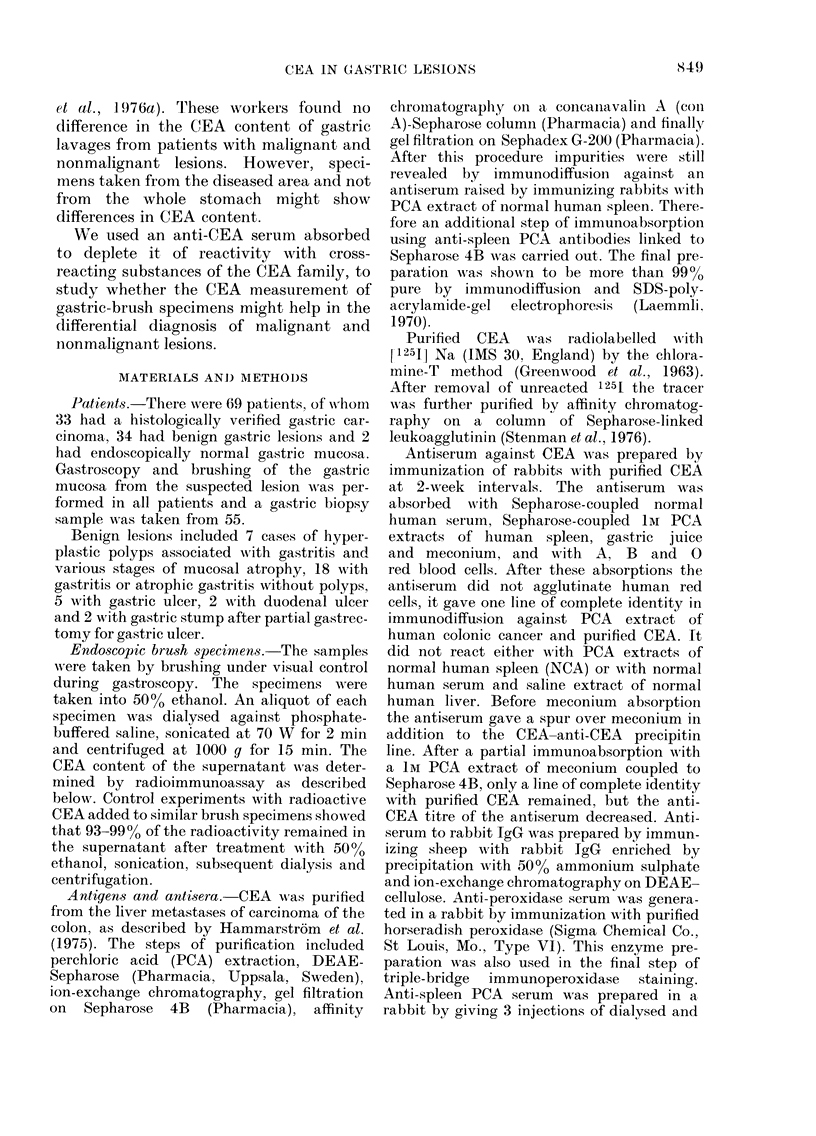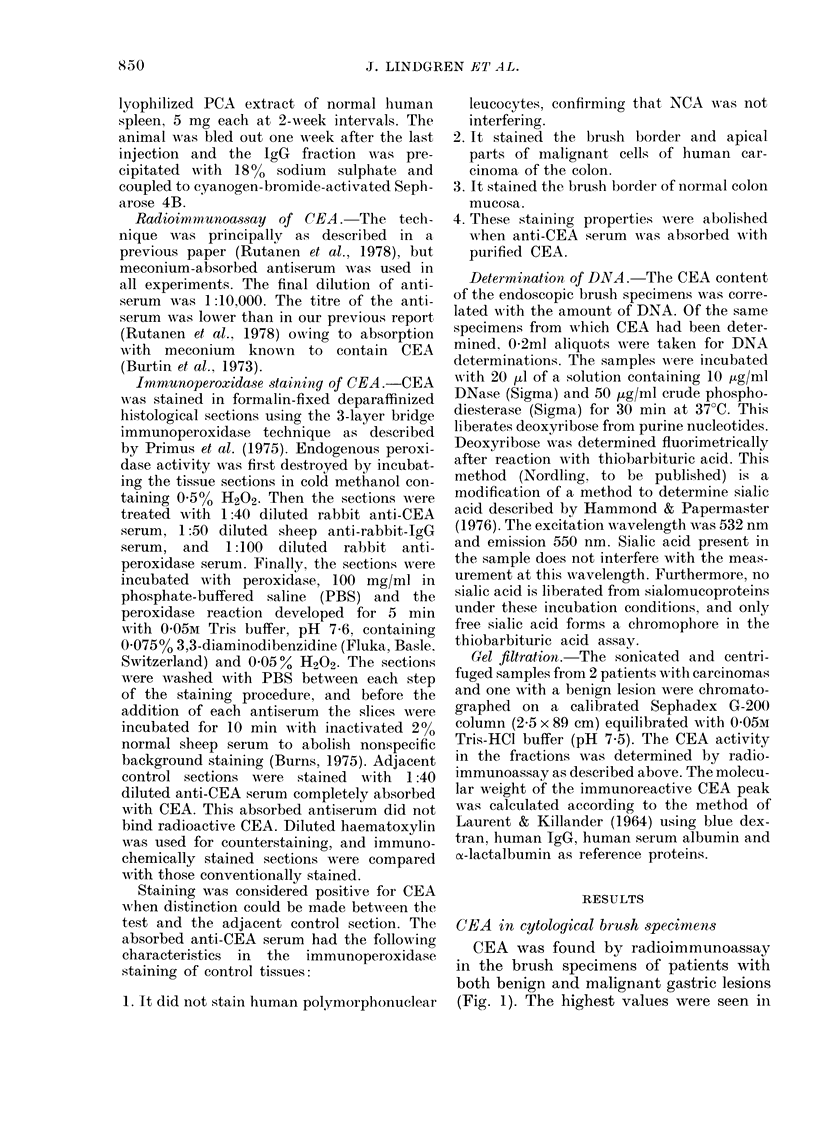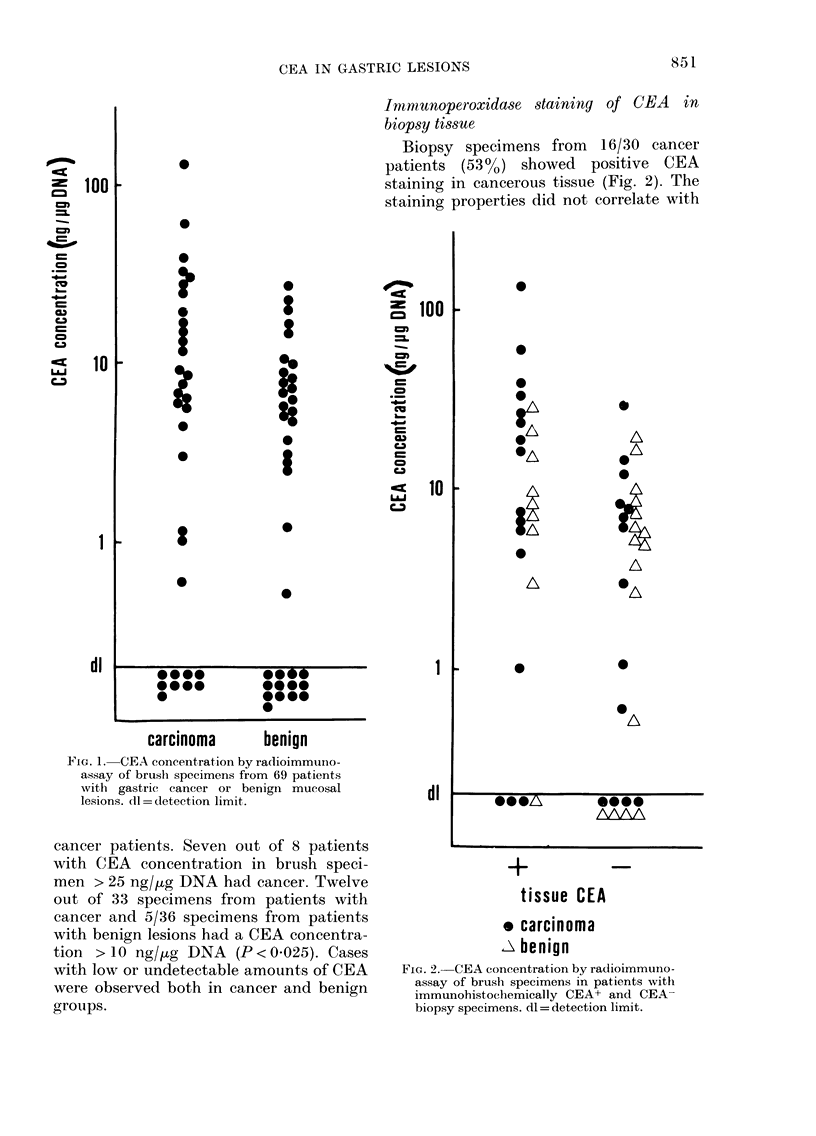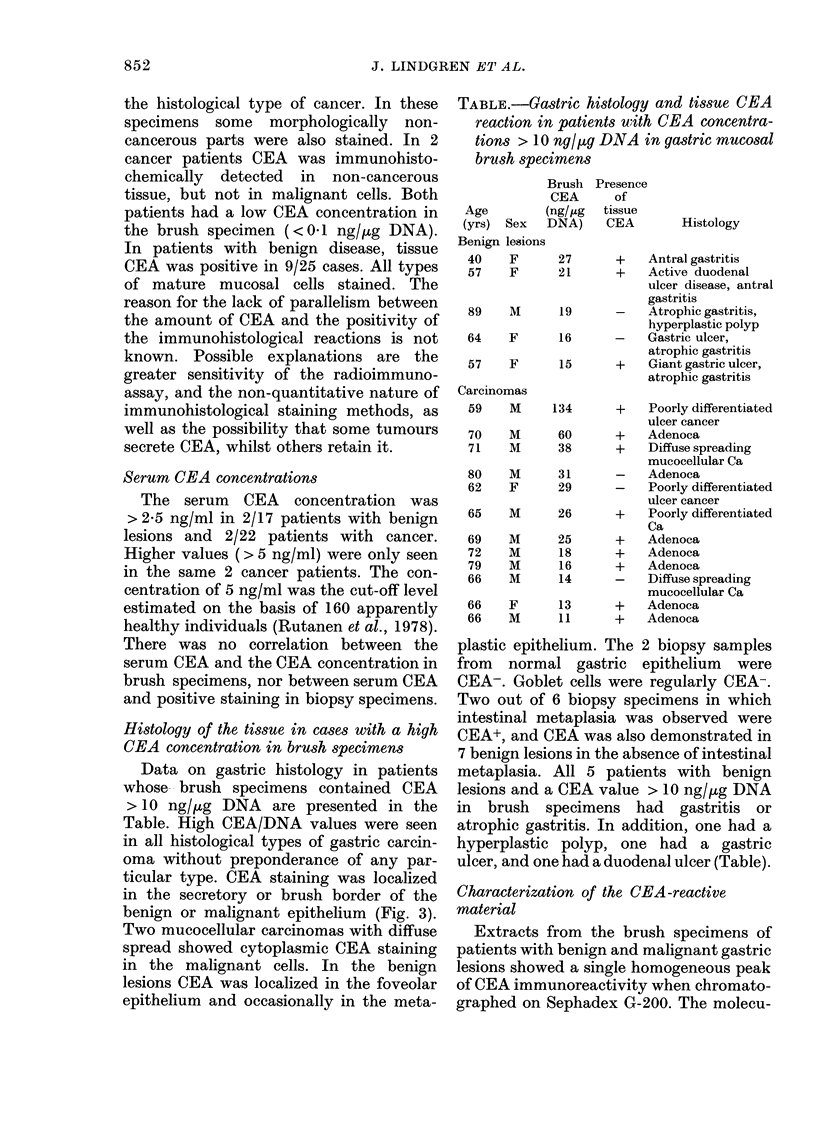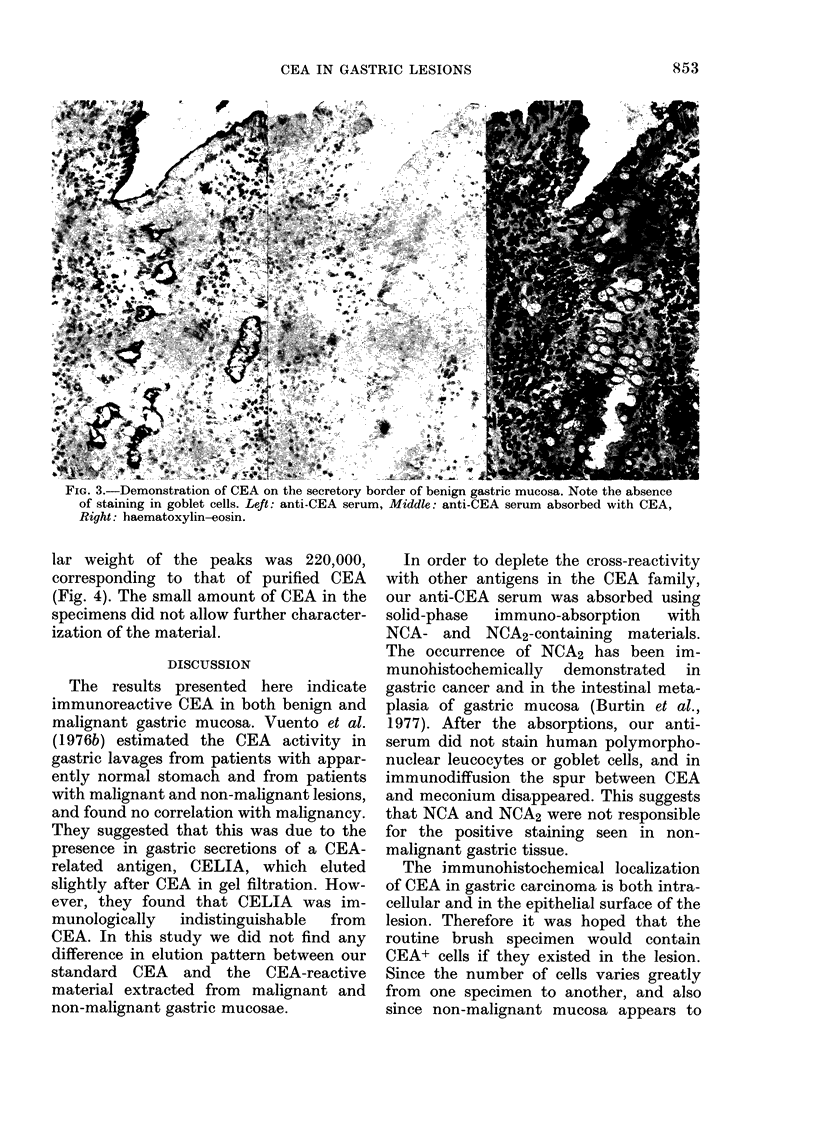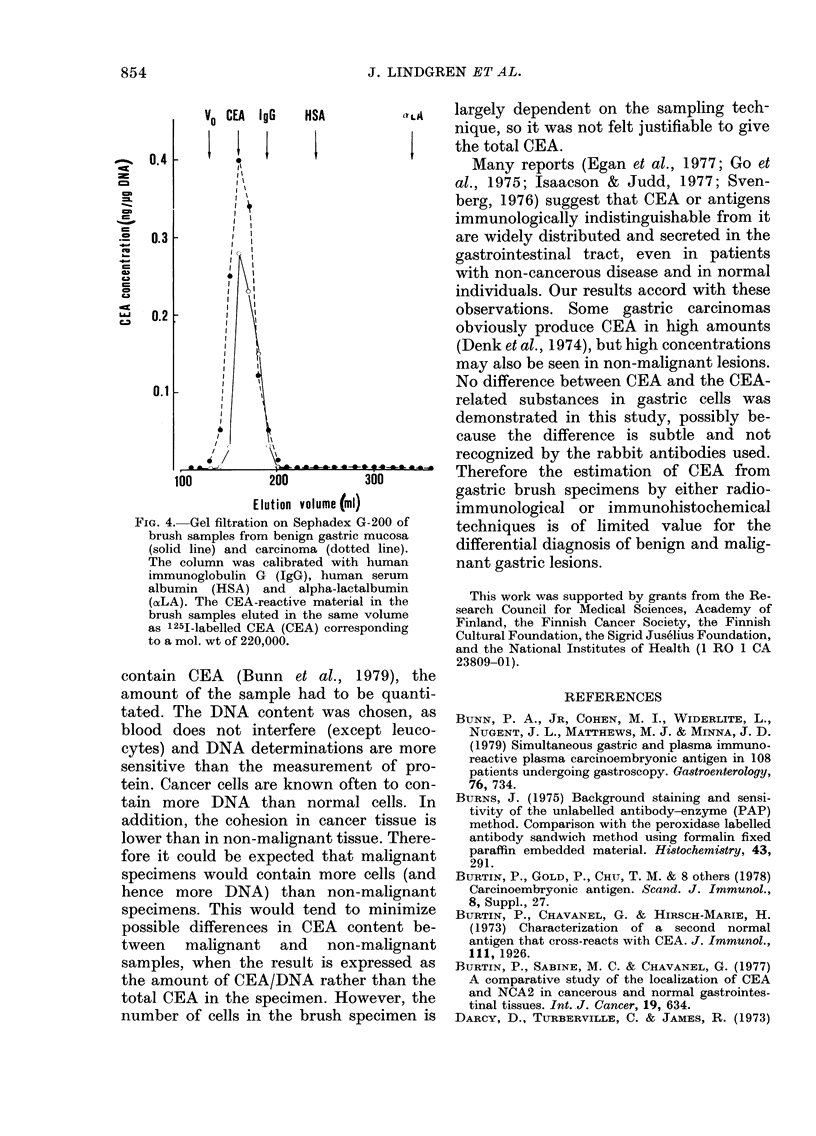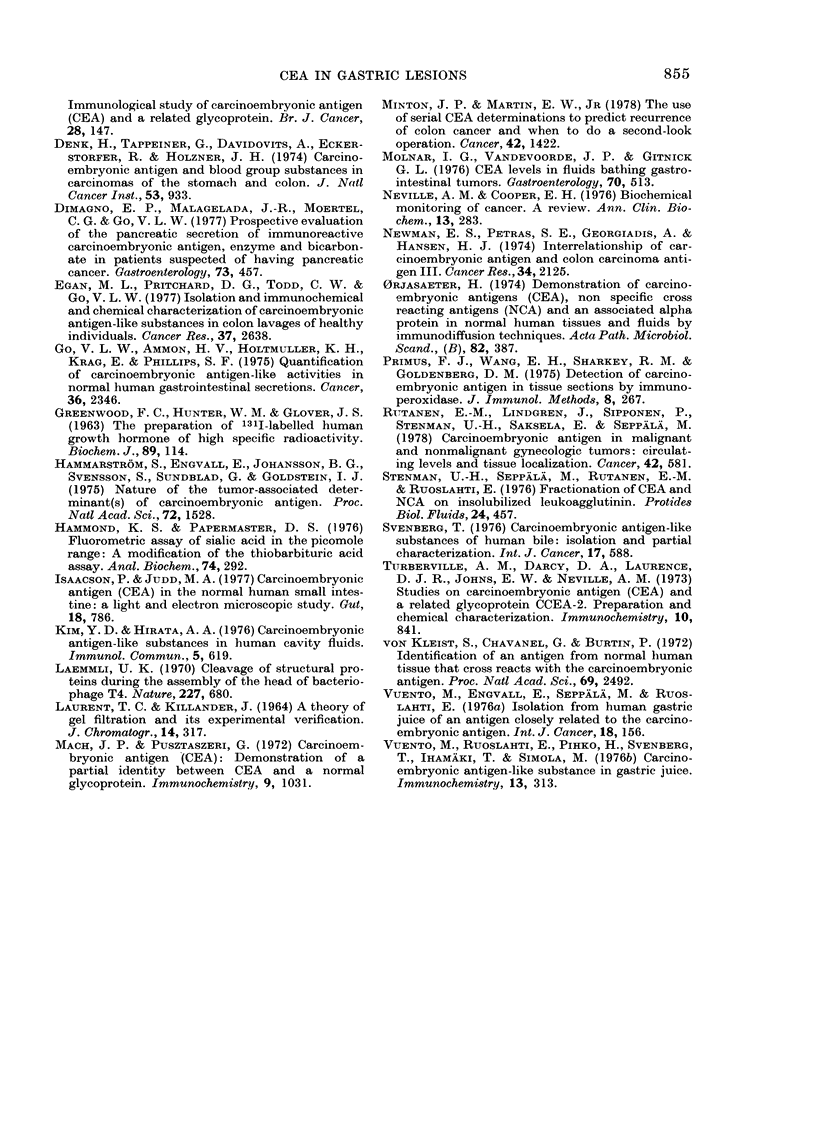# Carcinoembryonic antigen in endoscopic brush specimens from benign and malignant gastric lesions.

**DOI:** 10.1038/bjc.1979.275

**Published:** 1979-12

**Authors:** J. Lindgren, P. Sipponen, K. Seppälä, S. Tarpila, S. Nordling, T. Wahlström, M. Seppälä

## Abstract

**Images:**


*Br. J. Cancer* (1979) **40**, 848

### CARCINOEMBRYONIC ANTIGEN IN ENDOSCOPIC BRUSH SPECIMENS FROM BENIGN AND MALIGNANT GASTRIC LESIONS

J. LINDGREN*, P. SIPPONEN†, K. SEPPÄLÄ†, S. TARPILA‡, S. NORDLING§, T. WAHLSTRÖM§ AND M. SEPPÄLÄ*

*From the *Department of Bacteriology and Immunology, University of Helsinki, †Jorvi Hospital, Espoo, ‡Second Department of Medicine, University of Helsinki, and §Department of Pathology, University of Helsinki, Finland*

Received 30 May 1979 Accepted 21 August 1979

**Summary.—The measurement of carcinoembryonic antigen (CEA) in serum and endoscopic brush specimens was evaluated for the differential diagnosis of malignant and nonmalignant gastric disease. Brush specimens were studied from 33 patients with gastric cancer and 36 patients with benign gastric lesions or apparently normal gastric mucosa. Demonstrable CEA immunoreactivity was found by radioimmunoassay in brush specimens from 24/33 cancer patients (73%) and from 23/36 patients with benign lesions (64%). Patients with $CEA^+$ tissue in the immunoperoxidase test had somewhat higher CEA concentrations in the brush specimens than cases with $CEA^-$ biopsy tissue, although overlap was considerable. Thirty-five per cent of cancer patients had both a positive tissue CEA reaction and a CEA/DNA ratio >10 ng/μg, whilst patients with benign lesions had only 15% of positives by these criteria ($0{\cdot}01 > P > 0{\cdot}001$). The serum CEA concentration was above the upper normal level of 5 ng/ml in 2/39 patients, both of whom had gastric cancer. The CEA immunoreactive material from benign and malignant lesions eluted in gel filtration on Sephadex G-200 in the same volume as CEA purified from liver metastases of cancer of the colon, showing that a glycoprotein sharing immunological and physicochemical properties with CEA is present both in malignant and nonmalignant lesions of the gastric mucosa, and that there is considerable overlapping in the amount of CEA. The estimation of CEA in gastric-brush specimens is therefore of limited value in the differential diagnosis of benign and malignant gastric lesions.**

THE POSSIBILITY of using CEA estimation from plasma and other body fluids for the detection of cancer has been widely explored (for review see Burtin *et al.*, 1978). Whilst the plasma assay has turned out to be generally ineffective for the diagnosis of cancer, it still may be valuable for the detection of recurrent colorectal cancer (Minton & Martin, 1978; Neville & Cooper, 1976). Hopes have been raised that the measurement of CEA from secretions (Go *et al.*, 1975; DiMagno *et al.*, 1977) and effusion fluids (Molnar *et al.*, 1976; Kim *et al.*, 1976) might offer a more direct approach to the diagnosis of early malignant or premalignant lesions than the measurement of circulating CEA.

Several antigens cross-reacting with CEA have been identified and isolated from tissues and body fluids, *e.g.* NCA (von Kleist *et al.*, 1972), NGP (Mach & Pusztaszeri, 1972), CEX (Darcy *et al.*, 1973), CCEA-2 (Turberville *et al.*, 1973), CCAIII (Newman *et al.*, 1974), βE (Ørjasaeter, 1974), $NCA_2$ (Burtin *et al.*, 1973) and BGP I (Svenberg, 1976). NCA, NGP, CEX, CCEA-2, CCAIII and βE appear to be identical (Burtin *et al.*, 1978). A molecule closely related to CEA has been isolated from gastric juice (Vuento

Address for correspondence and reprints: Dr J. Lindgren, M.D., Department of Bacteriology and Immunology, University of Helsinki, Haartmaninkatu 3, 00290 Helsinki 29, Finland.

*et al.*, 1976*a*). These workers found no difference in the CEA content of gastric lavages from patients with malignant and nonmalignant lesions. However, specimens taken from the diseased area and not from the whole stomach might show differences in CEA content.

We used an anti-CEA serum absorbed to deplete it of reactivity with cross-reacting substances of the CEA family, to study whether the CEA measurement of gastric-brush specimens might help in the differential diagnosis of malignant and nonmalignant lesions.

### MATERIALS AND METHODS

*Patients.*—There were 69 patients, of whom 33 had a histologically verified gastric carcinoma, 34 had benign gastric lesions and 2 had endoscopically normal gastric mucosa. Gastroscopy and brushing of the gastric mucosa from the suspected lesion was performed in all patients and a gastric biopsy sample was taken from 55.

Benign lesions included 7 cases of hyperplastic polyps associated with gastritis and various stages of mucosal atrophy, 18 with gastritis or atrophic gastritis without polyps, 5 with gastric ulcer, 2 with duodenal ulcer and 2 with gastric stump after partial gastrectomy for gastric ulcer.

*Endoscopic brush specimens.*—The samples were taken by brushing under visual control during gastroscopy. The specimens were taken into 50% ethanol. An aliquot of each specimen was dialysed against phosphate-buffered saline, sonicated at 70 W for 2 min and centrifuged at 1000 *g* for 15 min. The CEA content of the supernatant was determined by radioimmunoassay as described below. Control experiments with radioactive CEA added to similar brush specimens showed that 93–99% of the radioactivity remained in the supernatant after treatment with 50% ethanol, sonication, subsequent dialysis and centrifugation.

*Antigens and antisera.*—CEA was purified from the liver metastases of carcinoma of the colon, as described by Hammarström *et al.* (1975). The steps of purification included perchloric acid (PCA) extraction, DEAE-Sepharose (Pharmacia, Uppsala, Sweden), ion-exchange chromatography, gel filtration on Sepharose 4B (Pharmacia), affinity chromatography on a concanavalin A (con A)-Sepharose column (Pharmacia) and finally gel filtration on Sephadex G-200 (Pharmacia). After this procedure impurities were still revealed by immunodiffusion against an antiserum raised by immunizing rabbits with PCA extract of normal human spleen. Therefore an additional step of immunoabsorption using anti-spleen PCA antibodies linked to Sepharose 4B was carried out. The final preparation was shown to be more than 99% pure by immunodiffusion and SDS-polyacrylamide-gel electrophoresis (Laemmli, 1970).

Purified CEA was radiolabelled with [$^{125}$I] Na (IMS 30, England) by the chloramine-T method (Greenwood *et al.*, 1963). After removal of unreacted $^{125}$I the tracer was further purified by affinity chromatography on a column of Sepharose-linked leukoagglutinin (Stenman *et al.*, 1976).

Antiserum against CEA was prepared by immunization of rabbits with purified CEA at 2-week intervals. The antiserum was absorbed with Sepharose-coupled normal human serum, Sepharose-coupled 1M PCA extracts of human spleen, gastric juice and meconium, and with A, B and O red blood cells. After these absorptions the antiserum did not agglutinate human red cells, it gave one line of complete identity in immunodiffusion against PCA extract of human colonic cancer and purified CEA. It did not react either with PCA extracts of normal human spleen (NCA) or with normal human serum and saline extract of normal human liver. Before meconium absorption the antiserum gave a spur over meconium in addition to the CEA–anti-CEA precipitin line. After a partial immunoabsorption with a 1M PCA extract of meconium coupled to Sepharose 4B, only a line of complete identity with purified CEA remained, but the anti-CEA titre of the antiserum decreased. Antiserum to rabbit IgG was prepared by immunizing sheep with rabbit IgG enriched by precipitation with 50% ammonium sulphate and ion-exchange chromatography on DEAE–cellulose. Anti-peroxidase serum was generated in a rabbit by immunization with purified horseradish peroxidase (Sigma Chemical Co., St Louis, Mo., Type VI). This enzyme preparation was also used in the final step of triple-bridge immunoperoxidase staining. Anti-spleen PCA serum was prepared in a rabbit by giving 3 injections of dialysed and lyophilized PCA extract of normal human spleen, 5 mg each at 2-week intervals. The animal was bled out one week after the last injection and the IgG fraction was precipitated with 18% sodium sulphate and coupled to cyanogen-bromide-activated Sepharose 4B.

*Radioimmunoassay of CEA.*—The technique was principally as described in a previous paper (Rutanen *et al.*, 1978), but meconium-absorbed antiserum was used in all experiments. The final dilution of antiserum was 1:10,000. The titre of the antiserum was lower than in our previous report (Rutanen *et al.*, 1978) owing to absorption with meconium known to contain CEA (Burtin *et al.*, 1973).

*Immunoperoxidase staining of CEA.*—CEA was stained in formalin-fixed deparaffinized histological sections using the 3-layer bridge immunoperoxidase technique as described by Primus *et al.* (1975). Endogenous peroxidase activity was first destroyed by incubating the tissue sections in cold methanol containing 0·5% $H_2O_2$. Then the sections were treated with 1:40 diluted rabbit anti-CEA serum, 1:50 diluted sheep anti-rabbit-IgG serum, and 1:100 diluted rabbit anti-peroxidase serum. Finally, the sections were incubated with peroxidase, 100 mg/ml in phosphate-buffered saline (PBS) and the peroxidase reaction developed for 5 min with 0·05M Tris buffer, pH 7·6, containing 0·075% 3,3-diaminodibenzidine (Fluka, Basle, Switzerland) and 0·05% $H_2O_2$. The sections were washed with PBS between each step of the staining procedure, and before the addition of each antiserum the slices were incubated for 10 min with inactivated 2% normal sheep serum to abolish nonspecific background staining (Burns, 1975). Adjacent control sections were stained with 1:40 diluted anti-CEA serum completely absorbed with CEA. This absorbed antiserum did not bind radioactive CEA. Diluted haematoxylin was used for counterstaining, and immunochemically stained sections were compared with those conventionally stained.

Staining was considered positive for CEA when distinction could be made between the test and the adjacent control section. The absorbed anti-CEA serum had the following characteristics in the immunoperoxidase staining of control tissues:

1. It did not stain human polymorphonuclear leucocytes, confirming that NCA was not interfering.
2. It stained the brush border and apical parts of malignant cells of human carcinoma of the colon.
3. It stained the brush border of normal colon mucosa.
4. These staining properties were abolished when anti-CEA serum was absorbed with purified CEA.

*Determination of DNA.*—The CEA content of the endoscopic brush specimens was correlated with the amount of DNA. Of the same specimens from which CEA had been determined, 0·2ml aliquots were taken for DNA determinations. The samples were incubated with 20 μl of a solution containing 10 μg/ml DNase (Sigma) and 50 μg/ml crude phosphodiesterase (Sigma) for 30 min at 37°C. This liberates deoxyribose from purine nucleotides. Deoxyribose was determined fluorimetrically after reaction with thiobarbituric acid. This method (Nordling, to be published) is a modification of a method to determine sialic acid described by Hammond & Papermaster (1976). The excitation wavelength was 532 nm and emission 550 nm. Sialic acid present in the sample does not interfere with the measurement at this wavelength. Furthermore, no sialic acid is liberated from sialomucoproteins under these incubation conditions, and only free sialic acid forms a chromophore in the thiobarbituric acid assay.

*Gel filtration.*—The sonicated and centrifuged samples from 2 patients with carcinomas and one with a benign lesion were chromatographed on a calibrated Sephadex G-200 column (2·5 × 89 cm) equilibrated with 0·05M Tris-HCl buffer (pH 7·5). The CEA activity in the fractions was determined by radioimmunoassay as described above. The molecular weight of the immunoreactive CEA peak was calculated according to the method of Laurent & Killander (1964) using blue dextran, human IgG, human serum albumin and α-lactalbumin as reference proteins.

### RESULTS

### *CEA in cytological brush specimens*

CEA was found by radioimmunoassay in the brush specimens of patients with both benign and malignant gastric lesions (Fig. 1). The highest values were seen in

FIG. 1.—CEA concentration by radioimmunoassay of brush specimens from 69 patients with gastric cancer or benign mucosal lesions. dl = detection limit.

cancer patients. Seven out of 8 patients with CEA concentration in brush specimen $>25$ ng/$\mu$g DNA had cancer. Twelve out of 33 specimens from patients with cancer and 5/36 specimens from patients with benign lesions had a CEA concentration $>10$ ng/$\mu$g DNA ($P<0{\cdot}025$). Cases with low or undetectable amounts of CEA were observed both in cancer and benign groups.

*Immunoperoxidase staining of CEA in biopsy tissue*

Biopsy specimens from 16/30 cancer patients (53%) showed positive CEA staining in cancerous tissue (Fig. 2). The staining properties did not correlate with

FIG. 2.—CEA concentration by radioimmunoassay of brush specimens in patients with immunohistochemically CEA$^{+}$ and CEA$^{-}$ biopsy specimens. dl = detection limit.

the histological type of cancer. In these specimens some morphologically non-cancerous parts were also stained. In 2 cancer patients CEA was immunohistochemically detected in non-cancerous tissue, but not in malignant cells. Both patients had a low CEA concentration in the brush specimen (< 0·1 ng/μg DNA). In patients with benign disease, tissue CEA was positive in 9/25 cases. All types of mature mucosal cells stained. The reason for the lack of parallelism between the amount of CEA and the positivity of the immunohistological reactions is not known. Possible explanations are the greater sensitivity of the radioimmunoassay, and the non-quantitative nature of immunohistological staining methods, as well as the possibility that some tumours secrete CEA, whilst others retain it.

*Serum CEA concentrations*

The serum CEA concentration was > 2·5 ng/ml in 2/17 patients with benign lesions and 2/22 patients with cancer. Higher values (> 5 ng/ml) were only seen in the same 2 cancer patients. The concentration of 5 ng/ml was the cut-off level estimated on the basis of 160 apparently healthy individuals (Rutanen *et al.*, 1978). There was no correlation between the serum CEA and the CEA concentration in brush specimens, nor between serum CEA and positive staining in biopsy specimens.

*Histology of the tissue in cases with a high CEA concentration in brush specimens*

Data on gastric histology in patients whose brush specimens contained CEA > 10 ng/μg DNA are presented in the Table. High CEA/DNA values were seen in all histological types of gastric carcinoma without preponderance of any particular type. CEA staining was localized in the secretory or brush border of the benign or malignant epithelium (Fig. 3). Two mucocellular carcinomas with diffuse spread showed cytoplasmic CEA staining in the malignant cells. In the benign lesions CEA was localized in the foveolar epithelium and occasionally in the metaplastic epithelium. The 2 biopsy samples from normal gastric epithelium were CEA⁻. Goblet cells were regularly CEA⁻. Two out of 6 biopsy specimens in which intestinal metaplasia was observed were CEA⁺, and CEA was also demonstrated in 7 benign lesions in the absence of intestinal metaplasia. All 5 patients with benign lesions and a CEA value > 10 ng/μg DNA in brush specimens had gastritis or atrophic gastritis. In addition, one had a hyperplastic polyp, one had a gastric ulcer, and one had a duodenal ulcer (Table).

TABLE.—*Gastric histology and tissue CEA reaction in patients with CEA concentrations > 10 ng/μg DNA in gastric mucosal brush specimens*

| Age (yrs) | Sex | Brush CEA (ng/μg DNA) | Presence of tissue CEA | Histology |
|---|---|---|---|---|
| Benign lesions | | | | |
| 40 | F | 27 | + | Antral gastritis |
| 57 | F | 21 | + | Active duodenal ulcer disease, antral gastritis |
| 89 | M | 19 | – | Atrophic gastritis, hyperplastic polyp |
| 64 | F | 16 | – | Gastric ulcer, atrophic gastritis |
| 57 | F | 15 | + | Giant gastric ulcer, atrophic gastritis |
| Carcinomas | | | | |
| 59 | M | 134 | + | Poorly differentiated ulcer cancer |
| 70 | M | 60 | + | Adenoca |
| 71 | M | 38 | + | Diffuse spreading mucocellular Ca |
| 80 | M | 31 | – | Adenoca |
| 62 | F | 29 | – | Poorly differentiated ulcer cancer |
| 65 | M | 26 | + | Poorly differentiated Ca |
| 69 | M | 25 | + | Adenoca |
| 72 | M | 18 | + | Adenoca |
| 79 | M | 16 | + | Adenoca |
| 66 | M | 14 | – | Diffuse spreading mucocellular Ca |
| 66 | F | 13 | + | Adenoca |
| 66 | M | 11 | + | Adenoca |

*Characterization of the CEA-reactive material*

Extracts from the brush specimens of patients with benign and malignant gastric lesions showed a single homogeneous peak of CEA immunoreactivity when chromatographed on Sephadex G-200. The molecu-

FIG. 3.—Demonstration of CEA on the secretory border of benign gastric mucosa. Note the absence of staining in goblet cells. *Left:* anti-CEA serum, *Middle:* anti-CEA serum absorbed with CEA, *Right:* haematoxylin–eosin.

lar weight of the peaks was 220,000, corresponding to that of purified CEA (Fig. 4). The small amount of CEA in the specimens did not allow further characterization of the material.

### DISCUSSION

The results presented here indicate immunoreactive CEA in both benign and malignant gastric mucosa. Vuento *et al.* (1976*b*) estimated the CEA activity in gastric lavages from patients with apparently normal stomach and from patients with malignant and non-malignant lesions, and found no correlation with malignancy. They suggested that this was due to the presence in gastric secretions of a CEA-related antigen, CELIA, which eluted slightly after CEA in gel filtration. However, they found that CELIA was immunologically indistinguishable from CEA. In this study we did not find any difference in elution pattern between our standard CEA and the CEA-reactive material extracted from malignant and non-malignant gastric mucosae.

In order to deplete the cross-reactivity with other antigens in the CEA family, our anti-CEA serum was absorbed using solid-phase immuno-absorption with NCA- and $NCA_2$-containing materials. The occurrence of $NCA_2$ has been immunohistochemically demonstrated in gastric cancer and in the intestinal metaplasia of gastric mucosa (Burtin *et al.*, 1977). After the absorptions, our antiserum did not stain human polymorphonuclear leucocytes or goblet cells, and in immunodiffusion the spur between CEA and meconium disappeared. This suggests that NCA and $NCA_2$ were not responsible for the positive staining seen in non-malignant gastric tissue.

The immunohistochemical localization of CEA in gastric carcinoma is both intracellular and in the epithelial surface of the lesion. Therefore it was hoped that the routine brush specimen would contain $CEA^+$ cells if they existed in the lesion. Since the number of cells varies greatly from one specimen to another, and also since non-malignant mucosa appears to

FIG. 4.—Gel filtration on Sephadex G-200 of brush samples from benign gastric mucosa (solid line) and carcinoma (dotted line). The column was calibrated with human immunoglobulin G (IgG), human serum albumin (HSA) and alpha-lactalbumin (αLA). The CEA-reactive material in the brush samples eluted in the same volume as $^{125}$I-labelled CEA (CEA) corresponding to a mol. wt of 220,000.

contain CEA (Bunn *et al.*, 1979), the amount of the sample had to be quantitated. The DNA content was chosen, as blood does not interfere (except leucocytes) and DNA determinations are more sensitive than the measurement of protein. Cancer cells are known often to contain more DNA than normal cells. In addition, the cohesion in cancer tissue is lower than in non-malignant tissue. Therefore it could be expected that malignant specimens would contain more cells (and hence more DNA) than non-malignant specimens. This would tend to minimize possible differences in CEA content between malignant and non-malignant samples, when the result is expressed as the amount of CEA/DNA rather than the total CEA in the specimen. However, the number of cells in the brush specimen is largely dependent on the sampling technique, so it was not felt justifiable to give the total CEA.

Many reports (Egan *et al.*, 1977; Go *et al.*, 1975; Isaacson & Judd, 1977; Svenberg, 1976) suggest that CEA or antigens immunologically indistinguishable from it are widely distributed and secreted in the gastrointestinal tract, even in patients with non-cancerous disease and in normal individuals. Our results accord with these observations. Some gastric carcinomas obviously produce CEA in high amounts (Denk *et al.*, 1974), but high concentrations may also be seen in non-malignant lesions. No difference between CEA and the CEA-related substances in gastric cells was demonstrated in this study, possibly because the difference is subtle and not recognized by the rabbit antibodies used. Therefore the estimation of CEA from gastric brush specimens by either radioimmunological or immunohistochemical techniques is of limited value for the differential diagnosis of benign and malignant gastric lesions.

This work was supported by grants from the Research Council for Medical Sciences, Academy of Finland, the Finnish Cancer Society, the Finnish Cultural Foundation, the Sigrid Jusélius Foundation, and the National Institutes of Health (1 RO 1 CA 23809–01).

### REFERENCES

BUNN, P. A., JR, COHEN, M. I., WIDERLITE, L., NUGENT, J. L., MATTHEWS, M. J. & MINNA, J. D. (1979) Simultaneous gastric and plasma immunoreactive plasma carcinoembryonic antigen in 108 patients undergoing gastroscopy. *Gastroenterology*, **76**, 734.

BURNS, J. (1975) Background staining and sensitivity of the unlabelled antibody–enzyme (PAP) method. Comparison with the peroxidase labelled antibody sandwich method using formalin fixed paraffin embedded material. *Histochemistry*, **43**, 291.

BURTIN, P., GOLD, P., CHU, T. M. & 8 others (1978) Carcinoembryonic antigen. *Scand. J. Immunol.*, **8**, Suppl., 27.

BURTIN, P., CHAVANEL, G. & HIRSCH-MARIE, H. (1973) Characterization of a second normal antigen that cross-reacts with CEA. *J. Immunol.*, **111**, 1926.

BURTIN, P., SABINE, M. C. & CHAVANEL, G. (1977) A comparative study of the localization of CEA and NCA2 in cancerous and normal gastrointestinal tissues. *Int. J. Cancer*, **19**, 634.

DARCY, D., TURBERVILLE, C. & JAMES, R. (1973)

Immunological study of carcinoembryonic antigen (CEA) and a related glycoprotein. *Br. J. Cancer*, **28**, 147.

Denk, H., Tappeiner, G., Davidovits, A., Eckerstorfer, R. & Holzner, J. H. (1974) Carcinoembryonic antigen and blood group substances in carcinomas of the stomach and colon. *J. Natl Cancer Inst.*, **53**, 933.

DiMagno, E. P., Malagelada, J.-R., Moertel, C. G. & Go, V. L. W. (1977) Prospective evaluation of the pancreatic secretion of immunoreactive carcinoembryonic antigen, enzyme and bicarbonate in patients suspected of having pancreatic cancer. *Gastroenterology*, **73**, 457.

Egan, M. L., Pritchard, D. G., Todd, C. W. & Go, V. L. W. (1977) Isolation and immunochemical and chemical characterization of carcinoembryonic antigen-like substances in colon lavages of healthy individuals. *Cancer Res.*, **37**, 2638.

Go, V. L. W., Ammon, H. V., Holtmuller, K. H., Krag, E. & Phillips, S. F. (1975) Quantification of carcinoembryonic antigen-like activities in normal human gastrointestinal secretions. *Cancer*, **36**, 2346.

Greenwood, F. C., Hunter, W. M. & Glover, J. S. (1963) The preparation of $^{131}$I-labelled human growth hormone of high specific radioactivity. *Biochem. J.*, **89**, 114.

Hammarström, S., Engvall, E., Johansson, B. G., Svensson, S., Sundblad, G. & Goldstein, I. J. (1975) Nature of the tumor-associated determinant(s) of carcinoembryonic antigen. *Proc. Natl Acad. Sci.*, **72**, 1528.

Hammond, K. S. & Papermaster, D. S. (1976) Fluorometric assay of sialic acid in the picomole range: A modification of the thiobarbituric acid assay. *Anal. Biochem.*, **74**, 292.

Isaacson, P. & Judd, M. A. (1977) Carcinoembryonic antigen (CEA) in the normal human small intestine: a light and electron microscopic study. *Gut*, **18**, 786.

Kim, Y. D. & Hirata, A. A. (1976) Carcinoembryonic antigen-like substances in human cavity fluids. *Immunol. Commun.*, **5**, 619.

Laemmli, U. K. (1970) Cleavage of structural proteins during the assembly of the head of bacteriophage T4. *Nature*, **227**, 680.

Laurent, T. C. & Killander, J. (1964) A theory of gel filtration and its experimental verification. *J. Chromatogr.*, **14**, 317.

Mach, J. P. & Pusztaszeri, G. (1972) Carcinoembryonic antigen (CEA): Demonstration of a partial identity between CEA and a normal glycoprotein. *Immunochemistry*, **9**, 1031.

Minton, J. P. & Martin, E. W., Jr (1978) The use of serial CEA determinations to predict recurrence of colon cancer and when to do a second-look operation. *Cancer*, **42**, 1422.

Molnar, I. G., Vandevoorde, J. P. & Gitnick G. L. (1976) CEA levels in fluids bathing gastrointestinal tumors. *Gastroenterology*, **70**, 513.

Neville, A. M. & Cooper, E. H. (1976) Biochemical monitoring of cancer. A review. *Ann. Clin. Biochem.*, **13**, 283.

Newman, E. S., Petras, S. E., Georgiadis, A. & Hansen, H. J. (1974) Interrelationship of carcinoembryonic antigen and colon carcinoma antigen III. *Cancer Res.*, **34**, 2125.

Ørjasaeter, H. (1974) Demonstration of carcinoembryonic antigens (CEA), non specific cross reacting antigens (NCA) and an associated alpha protein in normal human tissues and fluids by immunodiffusion techniques. *Acta Path. Microbiol. Scand.*, (*B*), **82**, 387.

Primus, F. J., Wang, E. H., Sharkey, R. M. & Goldenberg, D. M. (1975) Detection of carcinoembryonic antigen in tissue sections by immunoperoxidase. *J. Immunol. Methods*, **8**, 267.

Rutanen, E.-M., Lindgren, J., Sipponen, P., Stenman, U.-H., Saksela, E. & Seppälä, M. (1978) Carcinoembryonic antigen in malignant and nonmalignant gynecologic tumors: circulating levels and tissue localization. *Cancer*, **42**, 581.

Stenman, U.-H., Seppälä, M., Rutanen, E.-M. & Ruoslahti, E. (1976) Fractionation of CEA and NCA on insolubilized leukoagglutinin. *Protides Biol. Fluids*, **24**, 457.

Svenberg, T. (1976) Carcinoembryonic antigen-like substances of human bile: isolation and partial characterization. *Int. J. Cancer*, **17**, 588.

Turberville, A. M., Darcy, D. A., Laurence, D. J. R., Johns, E. W. & Neville, A. M. (1973) Studies on carcinoembryonic antigen (CEA) and a related glycoprotein CCEA-2. Preparation and chemical characterization. *Immunochemistry*, **10**, 841.

von Kleist, S., Chavanel, G. & Burtin, P. (1972) Identification of an antigen from normal human tissue that cross reacts with the carcinoembryonic antigen. *Proc. Natl Acad. Sci.*, **69**, 2492.

Vuento, M., Engvall, E., Seppälä, M. & Ruoslahti, E. (1976*a*) Isolation from human gastric juice of an antigen closely related to the carcinoembryonic antigen. *Int. J. Cancer*, **18**, 156.

Vuento, M., Ruoslahti, E., Pihko, H., Svenberg, T., Ihamäki, T. & Simola, M. (1976*b*) Carcinoembryonic antigen-like substance in gastric juice. *Immunochemistry*, **13**, 313.